# Core Clinical Phenotypes in Myotonic Dystrophies

**DOI:** 10.3389/fneur.2018.00303

**Published:** 2018-05-02

**Authors:** Stephan Wenninger, Federica Montagnese, Benedikt Schoser

**Affiliations:** Friedrich-Baur-Institute, Klinikum der Universität München, Munich, Germany

**Keywords:** myotonic dystrophies, DM1, DM2, phenotypes, myotonia, sleep disorders, repeat expansion diseases

## Abstract

Myotonic dystrophy type 1 (DM1) and type 2 (DM2) represent the most frequent multisystemic muscular dystrophies in adulthood. They are progressive, autosomal dominant diseases caused by an abnormal expansion of an unstable nucleotide repeat located in the non-coding region of their respective genes DMPK for DM1 and CNBP in DM2. Clinically, these multisystemic disorders are characterized by a high variability of muscular and extramuscular symptoms, often causing a delay in diagnosis. For both subtypes, many symptoms overlap, but some differences allow their clinical distinction. This article highlights the clinical core features of myotonic dystrophies, thus facilitating their early recognition and diagnosis. Particular attention will be given to signs and symptoms of muscular involvement, to issues related to respiratory impairment, and to the multiorgan involvement. This article is part of a Special Issue entitled “Beyond Borders: Myotonic Dystrophies—A European Perception.”

## Introduction

The genetic background of myotonic dystrophies type 1 and 2 (DM1 and DM2) is due to repeat expansions of unstable nucleotides in untranslated DNA regions causing mis-splicing of mRNAs, which affects almost all cells and organs of the human body. This sum of alterations leads to an extremely heterogeneous phenotype with musltisystemic involvement. Many findings and symptoms of DM1 and DM2 overlap, but important differences usually allow their prompt clinical distinction (Table 2). Both types of myotonic dystrophies represent the most common inherited muscle disorders in adulthood with regional variations in prevalence and incidence. In general, DM1 occurs more frequently than DM2, with some exeptions in northern and mid European countries such as Finland, Germany, and Czech Republic, where DM1 and DM2 are almost equally represented. Table 1 provides a useful summary of the country-specific prevalences (1–4).

**Table 1:** Country-specific prevalences of DM1 and DM2.

Country	Disease	Prevalence (×10^5^)	Reference
Croatia	DMs	18.1	(5)
Czech Republic	DM2	DM2 > DM1	(6)
Finland	DM2	10	(7)
Finland	DM2	54	(8)
Germany	DM2	DM1 = DM2	(9)
Israel	DM1	15.7	(10)
Italy	DMs	2.1	(11)
Italy	DM1	9.3	(12)
Italy	DM2	0.9–1	(13)
Italy	DM1	9.6–11.7	(13)
Japan	DMs	9.1	(14)
Spain, Mallorca	DMs	10.8	(15)
New Zeland, Otago	DMs	11.6	(16)
North Ireland	DMs	11.9	(17)
North Ireland	DMs	34	(18)
North UK	DM1	10.4	(19)
Quebec	DM1	210	(20)
Serbia, Belgrade	DM1	5.3	(21)
Taiwan	DM1	0.5	(22)

## Key Aspects in Myotonic Dystrophy Type 1

For DM1, there is a rough correlation between the expansion of CTG-repeats and the onset of symptoms as well as the severity of the disease; nevertheless predictions about the clinical features and the progression of the disease based on CTG-repeat size should be made very carefully (23, 24). 5 to 37 CTG-repeats are physiologic in healthy individuals. An expansion between 38 and 49 repeats does typically not cause any symptoms and reflects the premutation phenotype (3). The following four phenotypes are based on CTG-repeat sizes and onset of symtoms. It is important that phenotypes and CTG-repeat sizes do not show a linear and strict relationship and thus may overlap (25, 26):

- a mild phenotype with an expansion of 50–150 CTG-repeats,
- a classic phenotype with a wide span from mild to severe symptoms and an expansion of 50–1,000 CTG-repeats,
- a childhood/juvenile phenotype with early-onset and typically >800 CTG-repeats, and
- the most severe “congenital form” with usually >1,000 CTG-repeats.

CTG-repeats will expand in every following generation, and fully penetrant alleles occur with >50 CTG-repeats. This results in the so called anticipation, a clinical term describing an earlier onset with a more severe phenotype in the next generations (26). Furthermore, the repeat instability leads notably to premature aging of almost all organs, so DM1 may be counted among the progeroid diseases (27). The most typical appearance of DM1 is the “adult-onset” or “classic” phenotype with a CTG-repeat size ranging from 50 to <1,000. It is characterized by a distinctive combination of muscular symptoms, such as facial weakness, ptosis, grip myotonia, and distal muscle weakness with muscular atrophy. The classic phenotype is typically accompanied by extramuscular symptoms like cognitive impairment, cataracts, and diabetes mellitus. Nevertheless, as this multisystem disorder often presents with a high variability, some patients may primarily show only non-specific extramuscular symptoms like fatigue, daytime sleepiness, gastrointestinal symptoms, or cardiac conduction defects in an early stage of the disease, which could delay the diagnosis. Mildly affected patients with CTG-repeat sizes 50–100 may have normal or only minimally shortened lifespan (28). Because of comorbidities, such as cardiac and pulmonary complications, life expectancy is, however, reduced in about 70% of the patients with the classic phenotype (25).

## Special Aspects in Myotonic Dystrophy Type 2

DM2 (also referred to as proximal myotonic myopathy) is caused by the expansion of the tetranucleotide CCTG-repeat in the first intron of *CNBP* (cellular nucleic acid-binding protein), formerly known as zinc finger protein 9 (*ZNF9*) gene (29). Similar to DM1, these expansions are extremely unstable, causing widespread cellular abnormalities of mRNA splicing. In DM2, the expansion ranges from 75 to 11,000 with a mean of 5,000 CCTG-repeats. In contrast to DM1, there is no correlation between clinical phenotype and CCTG-repeat length and no anticipation has been observed (29, 30).

## Muscular Symptoms

### Muscular Weakness

**Figure 1:**
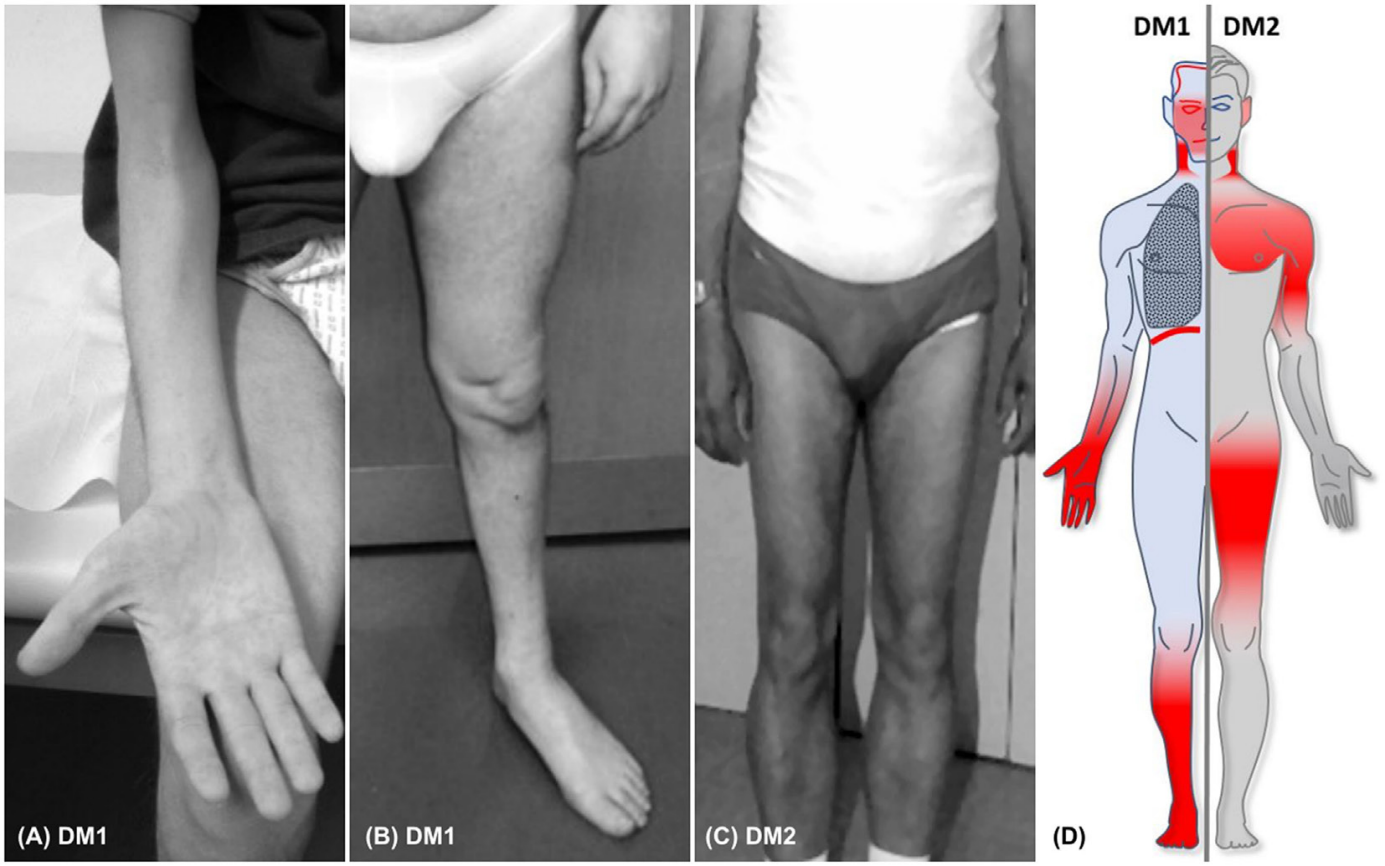
**(A,B)** Typical distal muscular atrophy in patients with DM1. **(C)** Atrophy of proximal muscles in a patient with DM2. **(D)** Figure illustrating the core phneotypes of DM1 (left) and DM2 (right). Regions of muscular involvement (weakness and atrophy) are highlighted in red.

The symptoms myotonia, muscular weakness, and muscular atrophy are the principal traits of DMs and gave the eponym for these two types of the disease. In DM1, patients present with characteristic distally predominant muscular atrophy and weakness mainly involving finger flexors, wrist flexors, and foot extensors (Figures 1A,B). The latter will cause foot drop and gait disturbance with repeated falls and injuries (31). In contrast to this, muscle weakness in DM2 is typically proximal and axial, affecting more consistently the neck flexors, hip flexors, and hip extensors (Figure 1C) (30, 32). This predominantly proximal muscular involvement has been documented also by MRI studies that showed an early degeneration of the erector spinae and gluteus maximus muscles (33, 34). Muscle weakness is one of the most frequently reported symptoms in DM1 (>45% of patients with adult phenotype) and DM2 (40–55% of patients) (26, 32). Figure 1D illustrates the predominantly affected muscle groups of patients with DM1 and DM2 (see Table 2 for differentiating DM1 and DM2).

**Table 2:** Core Clinical Symptoms helpful for differentiating DM1 and DM2.

		DM1	>DM2
Age of onset		Depends on CTG-repeat-size, in common first symptoms earlier than in DM2	30–40
Family history		Increasing severity of symptoms throughout generations (anticipation)	Variability in symptoms, but no evidence for anticipation
General appearance	Head	Forehead balding	
	Face	Myopathic face, temporal wasting, ptosis	
	Bulbar	Frequent: nasal/slurred speech, dysphagia	In some cases: dysphagia
Muscle	Weakness	Distal	Proximal and axial
	Myotonia	Handgrip, tongue	mild proximal
	Atrophy	Distal, early	Proximal, late
	Myalgia	Not typical, but may be secondary due to regional muscle imbalance	Predominant
Sleep disturbances		Central sleep apnea, obstructive sleep apnea, respiratory muscle weakness	Central sleep apnea
Central nervous system	Daytime sleepiness	In almost every patient	Frequent
	Concentration problems	Frequent	In some patients
	Hearing impaiment	Rare in adults, more frequent in congenital DM	Frequent
Diagnostics	Electromyography	Myotonic discharges in clinically affected and not affected muscles	Proximal, but can be absent

**Figure 2:**
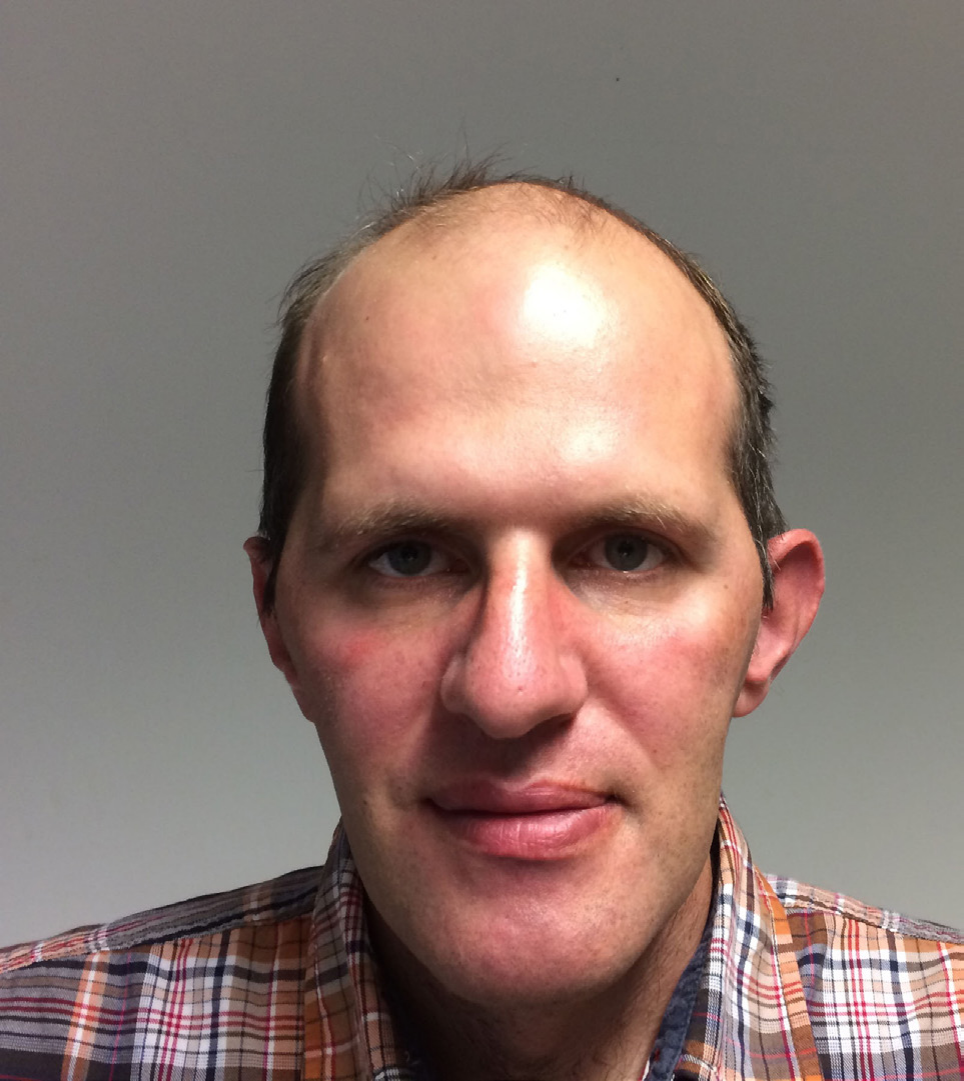
The characteristic face of a patient with DM1: long face, temporomandibular wasting, balding forehead.

The typical facial appearance of DM1 patients (“myopathic face”—“hatchet face”) is a prominent and early feature and is caused by weakness and atrophy of facial muscles and ptosis that might give the false impression of a tired, sad, or emotionless patient (35). Balding of the forehead and atrophying of the temporal muscle are often seen (Figure 2) and completes the overall picture of a patient with DM1. Severe weakness of orbiculari oculi muscles cause not only ptosis but also insufficient eyelid closure with risk of recurrent conjunctivitis. This facial muscle involvement is usually not seen in DM2 patients, thus it may help in differentiating DM1 from DM2 patients (Table 2).

Especially in patients with DM1, the speech can be nasal and slurred, due to the weakness of oropharyngeal muscles, sometimes causing chewing and swallowing difficulties.

### Myotonia

Myotonia is a more frequent symptom in DM1 mainly affecting the fingers (grip myotonia), the jaw, and the tongue (36). Clinically, a warm-up phenomenon is usually observed when myotonia improves with repeated contractions, which is mostly true for grip myotonia, but also for myotonia of the tongue and the jaw (37, 38). An increased excitability of muscle fibers is thought to be the cause for myotonia, leading to continuous discharges of repetitive action potentials after voluntary contraction or mechanical stimulation (39) in electromyography (EMG). These myotonic runs can be detected with EMG even in clinically unaffected muscles of DM1 patients, but can be rare or even be absent in DM2 (40). On a molecular basis, it has been suggested that myotonia is caused by mis-splicing of the chloride channel (*CLCN1*, ClC-1) due to misregulated MBNL1 and CUGBP1 (41). Recent studies have investigated the relationship between a central nervous system involvement and myotonia, suggesting that myotonia should no longer be considered as a solitary peripherally triggered muscular symptom (42, 43). In one fMRI study, higher cerebral blood oxygen level-dependent signals (BOLD) in specific primary and secondary motor areas were found during myotonia episodes. This was interpreted as a relationship between myotonia and high-order motor control areas (44). In another study, the severity of myotonia correlated with diffuse white matter alterations in specific primary and secondary motor areas (45). There are contradictory reports about the correlation between myotonia, grip strength, and CTG-repeat length (46, 47) for DM1 patients. Overall, grip strength correlates negatively with CTG-repeat length in most studies, but this is not necessarily true for myotonia. In one recent study, there was a statistically significant correlation between grip myotonia and CTG-repeat length, but this was not clinically meaningful and not predictive (47).

Myotonia seems to be usually mild to moderate or even absent in many DM2 patients, impacting only minimally their quality of life (36). However, its occurrence in different cohorts ranges between 24 and 75% (30, 32, 40). This variability is partly due to the discrepancy sometimes observed between a history of myotonia reported by patients and the clinical evidence of myotonic phenomenon, which is observed on neurological examination only in a minority of DM2 patients. Few patients may, however, display a severe myotonia and in some of these cases additional mutations in ion channel genes CLCN1 and SCN4A have been identified (48, 49). It is, therefore, advised to screen atypical cases with severe myotonia for mutation in these genes that act as phenotype modifier enhancing the myotonic phenomenon in DM2 (49). Particularly limb girdle myotonia is frequently neglected and underdiagnosed. With aging, the presence of myotonia gradually becomes less clinically relevant as it is overwhelmed by the gradually worsening of muscle weakness (32). This trend is also confirmed in studies assessing quality of life of DM2 patients, where significant predictors of worse QoL (quality of life) were older age, worse muscle strength, and higher level of fatigue (50).

### Musculoskeletal Pain or Myalgia

Musculoskeletal pain or myalgia may be present in some DM1 patients, but is less frequent in comparison to DM2 (Table 2). However, with the progression of the disease, a muscular imbalance due to weakness may occur and secondary complications such as regional myofascial pain or joint pain syndromes may develop even in DM1. In DM2, about 60% of patients complain of diffuse myalgia. These are usually exercise-related and worsen in cold temperatures (33, 51). Some patients consider pain as the most disabling symptom of the disease also because of its poor response to common analgesics (7). The pathophysiological mechanism of myalgia in DM2 is yet to be elucidated, but it is probably related to specific molecular changes occurring in the muscles of DM2 patients (52).

For adult-onset DM1, the first muscular symptoms can become apparent in early adulthood, but some patients may exhibit subtle symptoms like grip myotonia, ptosis or slurred speech in childhood. Patients with classic DM1 are typically diagnosed at around 30 years, but mildly affected patients with CTG-length 50–100 may present solely some slight myotonia or cataracts and may have their diagnosis delayed until they are around 40 years old (28). The clinical onset of DM2 typically occurs later than DM1, around the third to fourth decade; it may, however, often go unrecognized for several years due to only mild or unspecific clinical symptoms like myalgia or muscle cramps.

### Muscular Respiratory Symptoms

Respiratory muscle weakness will occur in a high percentage of the patients with DM1 in an early stage of the disease and chronic respiratory failure may develop (53). Expiratory muscles seem to be affected sooner than inspiratory muscles, resulting in early recurrent pneumonia due to a weak cough and insufficient airway clearance. The exact prevalence of respiratory insufficiency in DM1 is unclear because symptoms of nocturnal hypoventilation overlap with typical neuropsychological symptoms like fatigue, daytime sleepiness, and concentration difficulties (54). As both respiratory muscle weakness and cardiac symptoms account most for the reduced survival of the patients, repeated testing for early diagnosis is essential. A pure respiratory muscle weakness rarely occurs in DM2 and only about 6–15% of patients require non-invasive ventilation (55).

## Extramuscular Symptoms

### CNS Symptoms

Fatigue, daytime sleepiness, and concentration difficulties are frequently reported symptoms in DMs. In DM1, cognitive deficits were initially attributed to a low IQ or mental retardation, but recent studies show that this assumption was wrong for a large cohort of patients and mainly applies for cases of congenital myotonic dystrophy (CDM). In fact, for the classic phenotype of DM1, neuropsychological deficits are as variable as muscular symptoms, and even recent publications about the correlation of CTG-repeat size and neuropsychological deficits show contradictory study results (56–58). There seems to be a correlation between diffuse brain alterations in primarily white and secondary gray matter, linking the DM1 to the group of brain disconnection disorders (59). Caso et al. investigated 51 DM1 patients and found a correlation between changes in brain white matter and cognitive impairment (60). Cerebral white matter hyperintensities have been observed in both DM1 and DM2 patients, especially in those older than 40 years, but their clinical and functional significance still remains unclear (61–63). In a recent study about the educational profile of a large cohort of young DM1 patients, no significant differences compared to the healthy population were found (35), assuming that cognitive and concentration disturbances may occur later in the course of the disease in the context of a variable premature cognitive decline, as suggested by the study of Modoni et al. (56). Mild cognitive and behavioral symptoms are also present in DM2 patients. In particular, altered visuo-spatial and executive functions, reduced attention and flexibility of thinking, avoidant behavioral trait, and depression have been detected in these patients (63, 64). In many cases, neuropsychological disturbances jeopardize the ability to work and reduce the quality of life more than muscular symptoms.

Excessive daytime sleepiness, fatigue, and concentration difficulties may also be caused by central sleep disturbances or sleep apnea. Sleep-disordered breathing is one of the earliest manifestations and occurs in a high percentage of patients with DM1 (65), but overlapping symptoms of nocturnal hypoventilation and CNS symptoms may delay diagnosis and treatment. Chronic central sleep-disordered breathing has an impact on quality of life, morbidity, and mortality and should be assessed frequently in every patient with DM1 (54, 55, 66).

Until now, little is known about changes in CNS causing cognitive deficits and central sleep disrupted breathing. Almost every clinical study is conducted with the usually more affected DM1 patients, therefore data for DM2 patients are limited. On a molecular basis, MBNL1 and probably CELF may both be involved in CNS alterations, but little is known about molecular defects causing highly variable CNS symptoms in DM1 (42, 43). The above mentioned aspects lead to a discussion as to whether CNS dysfunction is caused by altered neurodevelopment, by neuro-dysfunction or by neurodegeneration within the definition of progeroid diseases (67). The hypothesis of a neurodegenerative disease is endorsed by findings of tau pathology and neurofibrillary degenerations, even if no correlations with CTG-repeat length were found (68). Overall, CNS dysfunction seems to be multifactorial.

### Eyes

The most frequent, early and typical extramuscular manifestation is the occurrence of early-onset cataract, observed in about 50–60% of patients (30, 32, 69). A medical history of cataract surgery in combination with muscular symptoms often leads to the diagnosis of DM, even in mildly affected patients without any sign of muscular impairment (70–72). The mechanisms underlying the pathophysiology of cataract in DMs are still largely unknown. At first, a potential effect of the CTG-mutation on the expression of neighboring genes such as SIX5 was considered in DM1 (73). But recent findings showed that SIX5 knock-out mice develop the nuclear type of cataract and not the posterior subcapsular/cortical type that are commonly observed in DMs. In addition, SIX5 is not adjacent to the DM2 repeat expansion so that this mechanism could not explain cataracts in DM2. More recent studies of global transcription performed on samples of lens epithelium in patients affected by DM1, DM2, and controls, identified a high similarity as regards the pattern of gene expression between DM1 and DM2 and hypothesized that common molecular mechanisms should be involved in cataract formation probably involving interferon signaling pathways (74, 75).

### Endocrine Symptoms

Endocrine dysfunctions such as diabetes, hypogonadism, and secondary hyperparathyroidism with decreased Vitamin-D levels are frequent in DMs and their occurrence increases with progression of the disease (76–78). A cross-sectional study on 68 DM1 patients showed at least one endocrine dysfunction in 44% at baseline and in 84% after 8 years (76). Diabetes mellitus, if not properly treated, may complicate and aggravate the clinical picture due to diabetic polyneuropathy with worsening of gait instability and distal weakness. Hyperparathyroidism may contribute to fatigue and muscle impairment (76). More rarely, abnormalities in growth hormone secretion and glucose intolerance may be observed (79).

### Hearing Impairment

Some degree of hearing loss has been described in DM2 since its first description (80). A recent systematic study on 56 Dutch and French DM2 patients then demonstrated that a mild to moderate hearing impairment was present in about 60% of examined patients. It is mostly a cochlear sensorineural hearing impairment which may be interpreted as an early presbycusis (81), well fitting in the interpretation of DMs as premature aging diseases. Similar features of cochlear impairment have also been described in some studies on DM1 patients (82).

### Cardiac Symptoms

In DMs, cardiac involvement is common. Cardiologic comorbidities include arrhythmias, atrial fibrillation, and conduction defects (e.g., AV-blocks) and often requires the implantation of pacemakers. Other infrequent manifestations are sudden death, heart failure, Brugada syndrome, ischemic heart disease, and mitral valve prolapse (83, 84). Dilated cardiomyopathies may also occur in some patients, but are not frequently found. Cardiac abnormalities in DM2 are similar to those observed in DM1 but occur less frequently. According to a recent observational case–control study on a large cohort of DM2/DM1 patients, it emerged that electrocardiographic abnormalities as PR > 200 ms and QRS > 100 ms were more frequent in DM1 (respectively, 31 and 48%) than DM2 patients (10 and 17%). Of those, 6 DM2 vs. 28 DM1 patients needed a pacemaker/implanted cardioverter (85). In the same study, echocardiography did not show any significant structural abnormalities but it was previously reported that a cardiomyopathy might occur in about 3% of DM2 patients. In DM1, the severity of cardiac involvement seems directly related with the size of CTG-expansion as recently studied by Chong-Nguyen et al. (9, 30, 85, 86). Atrial fibrillations and arrhythmias increase the risk of cerebral ischemia (87) and mortality and morbidity significantly depend on early cardiologic diagnosis and treatment (83).

### Gastrointestinal Symptoms

Along with elevations of creatine kinase, elevations of AST and ALT are frequent in patients with DM (88). In some cases, liver biopsies are performed because of these elevated “hepatic” enzymes without retrieving any pathologic result. The elevation of gamma-GT is suggested to be caused by contractions of bile canaliculi and bile ductules, whereas elevated levels of AST and ALT have their origin in skeletal muscle and go along with elevations of creatine kinase (89).

Alternating constipation, pseudo-constipation, bloating, and diarrhea are frequently reported symptoms in DM1, accompanied by stomach cramps, reflux, and regurgitation. They are caused by involvement of smooth and striated muscles and endocrine dysfunctions (90, 91). Swallowing problems are typical for DM1 patients and due to reduced oral transport that is caused by myotonia and weakness of the tongue. Dysphagia is caused by reduced swallowing reflex and reduced esophageal motility (92) which causes the major clinical problem by risk of aspiration. In conjunction with weakness of early affected expiratory muscles, this results in recurrent pneumonia and increased risk of death. A reduced or absent gastrointestinal peristaltic movement was earlier shown in radiological studies as well as delayed intestinal transits (93). Megacolon with the risk of ileus, volvulus and rupture, is a significant and life-threatening complication. Delayed emptying of the gall bladder may increase the risk for gallstones.

### Cancer

A higher incidence for neoplasms was found in several studies (28, 94, 95), most of them showed a predisposition in patients with DM1 for cancers such as skin cancer (like benign calcifying cutaneous tumors, pilomatricomas), thyroid, testicular, and prostate cancer. Because of the limited number of high-quality surveys and studies about the prevalence of cancer in DM1, further research is needed. A survey from the UK DM registry showed that 12.4% of the DM1/DM2 patients reported at least one benign tumor and 6.2% reported at least one malignant tumor with a high incidence of skin tumors (96), but there was no epidemiologic correlation with a non-DM-population.

### Peripheral Polyneuropathy

There is some debate as to whether peripheral neuropathy is a multisystemic manifestation of DMs or are caused by metabolic and endocrine dysfunctions. Its manifestation is not typical at early stages of the disease but may occur in about one-third of patients in later stages (97) of DM1 patients and contributes to balance impairment and increased risk of falls (31, 98). There were no significant correlations between age, duration of neuromuscular symptoms or CTG-repeat size (98, 99), suggesting that the affection of peripheral nerve system is secondary to metabolic and endocrine dysfunctions.

## Congenital Myotonic Dystrophy (CDM)

Patients with congenital DM1 have large CTG-expansions of more than 800, usually around 1,000. Characteristically, these large expansions are caused by maternal transmission, but CDM with paternal transmission is also known (23, 100–102). Clinically, CDM patients are severely affected and symptoms are often present before birth as polyhydramnios and reduced fetal movement. Hypotonia, generalized weakness, hyporeflexia, bilateral talipes, contractures, arthrogryposis, facial dysmorphia (carp mouth, ptosis, long neck and face, temporal muscle atrophy), and a weak cry are typical symptoms at birth or in the first days after delivery. Weak sucking and respiratory insufficiency often make ventilatory support unavoidable. Respiratory insufficiency is present in about 50% of newborns and is the main cause of dramatically reduced survival with a mortality rate of 30–40% (103). Infants who survive will typically reach their motor and cognitive milestones with some delay but might be able to walk independently. Similarly to DM1, a distal weakness is typical in CDM and a proximal involvement indicates a poor prognosis (104). Besides muscular symptoms, cognitive impairment, and neuropsychological disorders are the most common and variable manifestations in CDM. Symptoms range from intellectual impairment to selective cognitive impairment, apathy, and autism, as well as impaired attention, severe anxiety, and mood and depression syndromes (3, 102, 105–107). In the course of the disease, patients might require special schooling. In their third and fourth decades, patients may develop secondary complications, such as severe contractures, scoliosis, and worsening of cardiorespiratory symptoms (4).

## Childhood/Juvenile Onset DM1

The childhood and juvenile onset DM1 echoes the broad overlapping spectrum of symptoms of the congenital and the adult phenotypes. Commonly, there is an expansion of CTG-repeats of more than 800 repeats. First clinical symptoms may become apparent at age 1–10 for childhood onset and at age 10–20 for juvenile onset (3). Neurocognitive symptoms such as learning disability and learning difficulties are often prominent at age around 10 years and may become earlier apparent than muscular symptoms (107). In contrast to CDM, prenatal abnormalities or muscular symptoms right after delivery (neonatal hypotonia, sucking and swallowing difficulties and secondary dysmorphic features) are not typical, but a mild facial weakness or subtle facial dysmorphia may occur (3, 108). Early motor development is normal or only slightly delayed. Principal complaints in early childhood are speech and learning difficulties because of a mental handicap. At school, learning difficulties may become apparent and sometimes require special education. A study on 28 childhood-DM-patients showed that the full-scale IQ was significantly decreased (73.6) and 68% of the patients had repeated at least one school grade. 54% had additional psychiatric symptoms such as anxiety disorder, mood disorder, and attention-deficit-hyperactivity disorder (107). In adolescence, patients may show typical muscular and non-muscular symptoms of adult-onset DM1, e.g., like distal weakness, clinical myotonia, or gastrointestinal symptoms. Cardiologic symptoms, such as cardiac arrhythmias or cardiomyopathy, may occur, also leading to severe complications and sudden death (83). Life expectancy is not necessarily reduced, as long as core symptoms are recognized and treated sufficiently.

## Conclusion

Myotonic dystrophies represent the most variable clinical phenotypes, so treatment stratification is key for any modern therapeutic approach. We still need much more understanding of the signs and symptoms of DM patients in correlation to their molecular origins.

## Author Contributions

SW: review of publications, writing, critical revision of manuscript for intellectual content, and final approval of the manuscript. FM: review of publications, writing, and critical revision of manuscript for intellectual content. BS: critical revision of manuscript for intellectual content.

## Conflict of Interest Statement

The authors declare that the research was conducted in the absence of any commercial or financial relationships that could be construed as a potential conflict of interest.
